# Status check: next-generation sequencing for infectious-disease diagnostics

**DOI:** 10.1172/JCI178003

**Published:** 2024-02-15

**Authors:** Kyle G. Rodino, Patricia J. Simner

**Affiliations:** 1 Department of Pathology and Laboratory Medicine, Perelman School of Medicine, University of Pennsylvania, Philadelphia, Pennsylvania, USA.; 2 Department of Pathology, Johns Hopkins School of Medicine, Baltimore, Maryland, USA.

## Abstract

<p>Next-generation sequencing (NGS) applications for the diagnosis of infectious diseases provides advantages over traditional microbiologic methods, though challenges of pathogen detection and antimicrobial resistance continue.</p>

Next-generation sequencing (NGS) applications for the diagnostics of infectious diseases has demonstrated great potential with three distinct approaches: whole-genome sequencing (WGS), targeted NGS (tNGS), and metagenomic NGS (mNGS, also known as clinical metagenomics). These approaches provide several advantages over traditional microbiologic methods, though challenges still exist.

## Whole-genome sequencing

**Figure 1:**
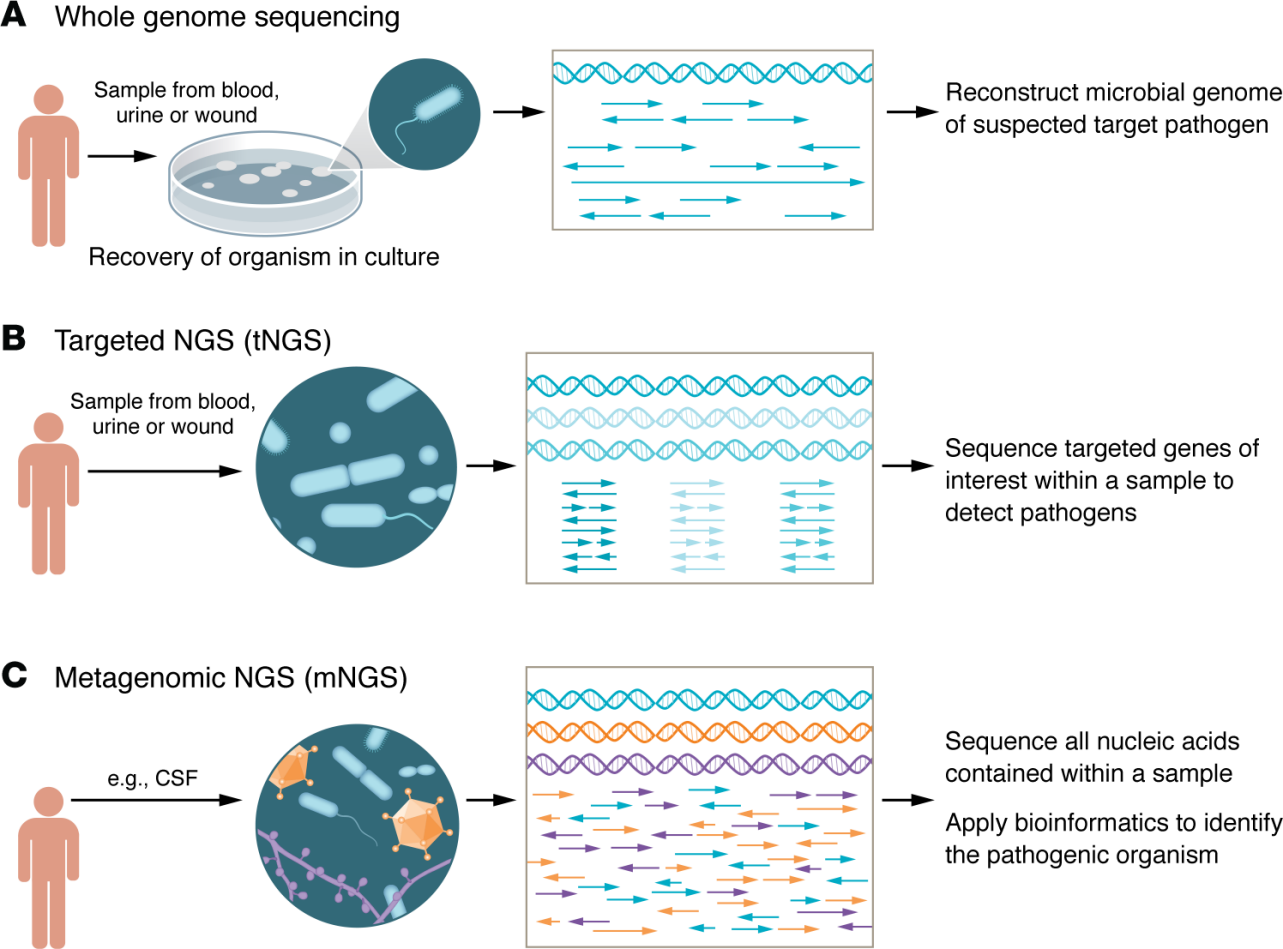
NGS applications can be used in the diagnosis of infectious diseases. (**A**) Microbes grown from patient samples provide microbial DNA for WGS in which millions of fragments are read in parallel and assembled to reconstruct the microbial genome. WGS is especially useful for the identification of pathogens associated with hospital outbreaks and detection of AMR genes. (**B**) tNGS directly amplifies genes from a clinical specimen prior to sequencing. This technique often focuses on genes that are shared among all members of a microbial kingdom, such as the 16S rRNA gene for bacteria. The sequencing of amplified products allows for detection and identification of the composition of the targeted microorganisms in the specimen. Expanded applications can assess thousands of genes in parallel, covering pathogens and AMR targets. (**C**) mNGS involves sequencing all nucleic acids contained within a sample (e.g. CSF), including those derived from the host, microbes, and even contaminating nucleic acid. mNGS shows promise in detecting rare pathogens in which diagnostics are unavailable, unsuspected pathogens based on the patients’ symptoms with conditions that affect routine testing results, and in situations with insidious organisms.

In whole-genome sequencing (WGS), millions of fragments of microbial DNA are read in parallel. These overlapping reads are then bioinformatically assembled for complete reconstruction of the microbial genome, permitting enhanced pathogen identification and discovery (Figure 1A). Such detailed genomic information is finding use in clinical laboratories to support epidemiological investigations of hospital outbreaks and to track the genetic determinants of antimicrobial resistance (AMR). Economic analyses have demonstrated the value of prospective WGS over traditional reactive approaches to identify and contain hospital-acquired infection clusters (1, 2). Furthermore, data from the whole genome and/or resistome (all AMR genes) of a bacterial pathogen combined with machine-learning approaches have enabled predictions of the phenotypic susceptibility profile — with similar accuracy as traditional growth-based approaches (3). Applying rapid WGS could provide more timely results to guide patient management (4).

## Targeted next-generation sequencing

With targeted next-generation sequencing (tNGS), the target of interest, commonly a gene shared among all members of a microbial kingdom, is amplified direct from a clinical specimen prior to sequencing (Figure 1B). The amplified products are then sequenced, allowing for detection and identification of the composition of the targeted microorganisms in the specimen. The approach is colloquially referred to as “broad range” PCR with sequencing, and the most common example is that which targets the 16S rRNA gene to allow profiling of individual bacterial species from clinical samples. However, it can also be applied in a similar manner with alternative targets to fungi and mycobacteria. In an expanded application, large tNGS panels can assess thousands of genes in parallel, covering pathogens and AMR targets with enhanced sensitivity, theoretically, due to the targeted nature of the assays directly from patient specimens (5). Importantly, curated microbial genome databases serve as the basis for analysis and interpretation of other direct-from-specimen sequencing approaches. Thus, the accuracy of tNGS and metagenomic next-generation sequencing (mNGS) often relies on the need for high quality microbial whole-genome sequences.

## mNGS

Contrary to tNGS, mNGS does not require a suspected target and derives most of its value from an untargeted, sometimes termed “shotgun”, approach to pathogen detection. mNGS involves sequencing all nucleic acids contained within a sample, including those derived from the host, microbes, and even contaminating nucleic acid (Figure 1C). Processes to remove the unwanted nucleic acids presequencing or unwanted reads postsequencing can be applied, but in the end this method allows for direct detection of pathogen reads among a sea of total sequencing data. Therefore, mNGS is a hypothesis-free method to search for any and all possible pathogens (e.g., bacterial, viral, fungal, and parasitic) — and, if lucky, AMR genes — in a sample concomitantly. mNGS has found early success in detecting rare pathogens where targeted diagnostics are not available, in detecting pathogens presenting in abnormal manners, in detecting pathogens where immunocompromising conditions affect performance of routine testing, and in earlier detection of fastidious or insidious organisms. To date, the most widely studied and available mNGS assays look for pathogens in cerebrospinal fluid (CSF) and plasma, with many cases, case series, and observational outcome studies supporting its use (6–15). Offerings geared toward other sample types are expanding and novel use cases continue to be described, including the optimal timing of testing, specific patient populations, or clinical syndromes. The continued development of mNGS provides the opportunity for its use a precision-medicine tool that reveals additional information about the host response and microbiome in addition to details about pathogen identification, virulence factors, and resistance genes.

## Limitations and challenges in direct pathogen detection

As with all molecular methods for direct pathogen detection, tNGS and mNGS are limited by the presence of the organisms in a clinical sample at the time of collection. This is true in the context of pathogens found in low burden, such as *Borrelia burgdorferi* in plasma. A study evaluating potential clinical utility of plasma cell-free DNA (cfDNA) mNGS for acute Lyme disease diagnosis showed enhanced ability to detect *B*. *burgdorferi* (16). Increased sensitivity is derived from the ability to detect *B*. *burgdorferi* from any uniquely identifiable cfDNA fragment by mNGS, not limiting detection to the fragment targeted in *B*. *burgdorferi*–specific PCRs. However, as acknowledged in the manuscript, many of the detections were attributed to use of an investigational reporting threshold, which would have been missed by the clinically available protocol (16). Improvement in sensitivity or adjustment in validated clinical reporting would be needed to realize the benefit of mNGS in this application. A similar situation of inconsistent detection is found with neuroinvasive viruses with temporal viremia. By the onset of neurologic manifestations, the window of virus presence in CSF has passed in most cases, and mNGS is often negative. This limitation, caused by viral dynamics, was born out in an investigation of patients with acute flaccid myelitis where mNGS detected enterovirus RNA in only one case, while panviral serology was able to detect EV-specific antibodies in many cases (17). It has also been shown that mNGS of plasma is not always a reliable surrogate for testing of the locally infected area. In a small study comparing paired samples, mNGS detected the pathogen in 3 of 9 plasma samples, while detecting the pathogen in 8 of 9 local body fluids (18). Intuitively, reads corresponding to the pathogen were present in much higher abundance in the body fluid compared with plasma, highlighting the importance of sample collection in comprehensive mNGS evaluation. As direct-from-specimen NGS approaches are complex, high cost, and labor-intensive approaches, their use has largely been applied as a last-resort diagnostic when standard-of-care methods are unrevealing. However, the appropriate timing of testing in the care pathway remains poorly understood, with some studies suggesting that early testing may reduce healthcare expenditures (19, 20). If direct-from-specimen NGS assays are applied earlier in care and more readily reveal the infectious etiology, there is potential to reduce overall healthcare expenditures, similar to WGS for outbreak investigations.

The power of mNGS to detect pathogen nucleic acid in an untargeted fashion also creates the need to evaluate results in the clinical context. There is risk in detecting contaminating nucleic acid, whether originating from collection, handling, or assay reagents, or detecting residual organisms unrelated to the current process. For instance, approximately 20% of people in the healthy control group used to validate the specificity of a cfDNA plasma metagenomic assay were positive and reflected commensal microbiota (6). Further, some of the most common reagent contaminants are also known pathogens such as *Enterobacterales, Staphylococcus* species, and *Pseudomonas* species, further confounding interpretation of results (21). The reporting of these false-positive detections can create challenges for treatment teams tasked with adjudication and may lead to erroneous diagnosis or unnecessary treatment. Auspiciously, well-designed experimental controls can be included in the assay design to try to account for most of these experimental contaminants. However, computational contaminants can be more difficult to discern, highlighting the need for well-curated databases utilized for analysis. mNGS involves first removing host reads and then comparing all remaining RNA or DNA reads from a patient specimen to microbial genomes to identify a cause of infection. Lack of the microorganism(s) or even at minimum, taxonomically related relatives, in a database can lead to false-negative results. Computational contaminants include small amounts of DNA not derived from the organism of interest that get unknowingly assembled into a whole genome (21). Greater than 3,000 published microbial genomes have been previously reported to contain small fragments of human genome contaminants (22). Conversely, microbial DNA can be assembled into human whole-genome sequences. These computational contaminants can lead to spurious associations between microbes and disease. For example, one study evaluating 5,000 human genomes identified 50 bacteria that were more common among males than among females, supporting sex-associated bacteria (23). However, further analysis led them to conclude the association was false and caused by computation contamination of the bacterial genome reference sequences with portions of the previously incomplete human Y chromosome (24). From a mNGS standpoint, experimental contaminants can lead to false-negative results, such as removal of microbial reads that are assembled into the human genome, or false-positive results, when human DNA results in an inaccurate association to a microbial hit due to assembly of human reads into microbial genomes. Thus, accurate genomes are required to improve diagnostic accuracy.

## Limitations and challenges in direct AMR detection

Direct detection of AMR genes from patient specimens can be accomplished by a tNGS approach or by chance using a mNGS approach. tNGS approaches have theoretically enhanced sensitivity over untargeted approaches, whereas tNGS is limited by the assay targets. AMR detection by mNGS depends on the type of AMR determinant — such as acquired genes of several hundred base pairs in length versus a single nucleotide polymorphism (SNP) in a gene — abundance of the pathogen, and composition of the specimen. Intuitively, you have a higher likelihood of detecting an entire acquired resistance gene over a SNP-based mutation that leads to resistance. In samples with high-abundance monomicrobial pathogens, detection of AMR genes has higher accuracy due the presence of a more complete genome and higher likelihood of detecting the resistome of the organism among the detected microbial reads over lower abundance pathogens with fragmented genomes. Unlike mNGS for pathogen detection that can be accomplished with only a hand full of unique genes spanning the genome, accurate AMR detection requires more coverage of the genome. In one case of a previously treated, culture-negative lung biopsy specimen, mNGS was utilized to identify an uncultivatable high-abundance pathogen, detect associated AMR, and predict the phenotype to successfully guide therapy (18). It is important to note that there is poor negative predictive value, as the lack of detection of AMR does not rule out the presence of an AMR gene. Also, for polymicrobial specimens, association of the AMR gene with a particular pathogen is difficult as it can be impossible to link the AMR marker to a specific organism, unless long read sequencing provides genetic context to the AMR gene (3). Furthermore, the AMR gene cannot be linked to one pathogen over another if multiple pathogens known to harbor the AMR gene are detected, or for cases in which benign microbiota harbor AMR genes, which is a scenario that can lead to predictions of false resistance. Similar to pathogen detection, the detection of AMR genes and SNPs rely on the databases used to identify them, and one study suggests that multiple databases should be used (25). A study evaluating a broad targeted AMR panel from bronchoalveolar lavage specimens, AMR markers were detected in the great majority of the samples (136 of 201 [68%]) (5). However, these AMR markers could only be associated with a putative pathogen in 15 samples. Additionally, full or partial agreement between AMR detection and phenotypic AST was found in only half of the pathogens with associated AMR. AMR associations were made for consequential resistance organisms, such as extended-spectrum β-lactamase–producing (ESBL-producing) *Escherichia coli*, vancomycin-resistant enterococci, methicillin-resistant *Staphylococcus aureus,* and *Mycobacterium tuberculosis*. However, it missed a *bla*_KPC_ carbapenemase gene associated with a KPC-producing *Klebsiella pneumoniae*, and, in one instance, the *bla*_CTX-M-15_ ESBL gene was detected and associated with both *E*. *coli* and *Pseudomonas aeruginosa* where phenotypic testing revealed it belonged only to the *Escherichia coli* isolate (5). Thus, it appears that AMR detection will require targeted or enrichment approaches via direct-from-specimen, NGS-based approaches. Otherwise, there is a need for methods with the capability of enriching for microbial reads and permitting WGS assembly directly from the specimen to allow higher accuracies of prediction.

## Status in progress

As we observe the further introduction of NGS-based diagnostics for infectious diseases into clinical microbiology laboratories, it is important to understand the various approaches and associated limitations to these approaches. It is imperative that the end users of these tests understand how methodologic variants to these approaches can affect the interpretation of the results for clinical care. Currently, most NGS assays are limited to academic medical centers or commercial laboratories. As we start to see further automation of methods, automated analytic programs to analyze results, and a reduction in costs, we will see further uptake of the technology to guide patient care akin to the uptake of PCR modalities in the early 2000s.

## Version 1 02/15/2024

Electronic publication

## Version 2 02/15/2024

Formatting of opening paragraph corrected
